# Role of the site of synaptic competition and the balance of learning forces for Hebbian encoding of probabilistic Markov sequences

**DOI:** 10.3389/fncom.2015.00092

**Published:** 2015-07-21

**Authors:** Kristofer E. Bouchard, Surya Ganguli, Michael S. Brainard

**Affiliations:** ^1^Life Sciences and Computational Research Divisions, Lawrence Berkeley National LaboratoryBerkeley, CA, USA; ^2^Department of Applied Physics, Stanford UniversityStanford, CA, USA; ^3^Department of Physiology, University of California, San Francisco and Center for Integrative Neuroscience, University of California, San FranciscoSan Francisco, CA, USA; ^4^Howard Hughes Medical InstituteChevy Chase, MD, USA

**Keywords:** Hebbian plasticity, pre/post-synaptic, probability, sequences, birdsong

## Abstract

The majority of distinct sensory and motor events occur as temporally ordered sequences with rich probabilistic structure. Sequences can be characterized by the probability of transitioning from the current state to upcoming states (forward probability), as well as the probability of having transitioned to the current state from previous states (backward probability). Despite the prevalence of probabilistic sequencing of both sensory and motor events, the Hebbian mechanisms that mold synapses to reflect the statistics of experienced probabilistic sequences are not well understood. Here, we show through analytic calculations and numerical simulations that Hebbian plasticity (correlation, covariance, and STDP) with pre-synaptic competition can develop synaptic weights equal to the conditional forward transition probabilities present in the input sequence. In contrast, post-synaptic competition can develop synaptic weights proportional to the conditional backward probabilities of the same input sequence. We demonstrate that to stably reflect the conditional probability of a neuron's inputs and outputs, local Hebbian plasticity requires balance between competitive learning forces that promote synaptic differentiation and homogenizing learning forces that promote synaptic stabilization. The balance between these forces dictates a prior over the distribution of learned synaptic weights, strongly influencing both the rate at which structure emerges and the entropy of the final distribution of synaptic weights. Together, these results demonstrate a simple correspondence between the biophysical organization of neurons, the site of synaptic competition, and the temporal flow of information encoded in synaptic weights by Hebbian plasticity while highlighting the utility of balancing learning forces to accurately encode probability distributions, and prior expectations over such probability distributions.

## Introduction

**Figure 1:**
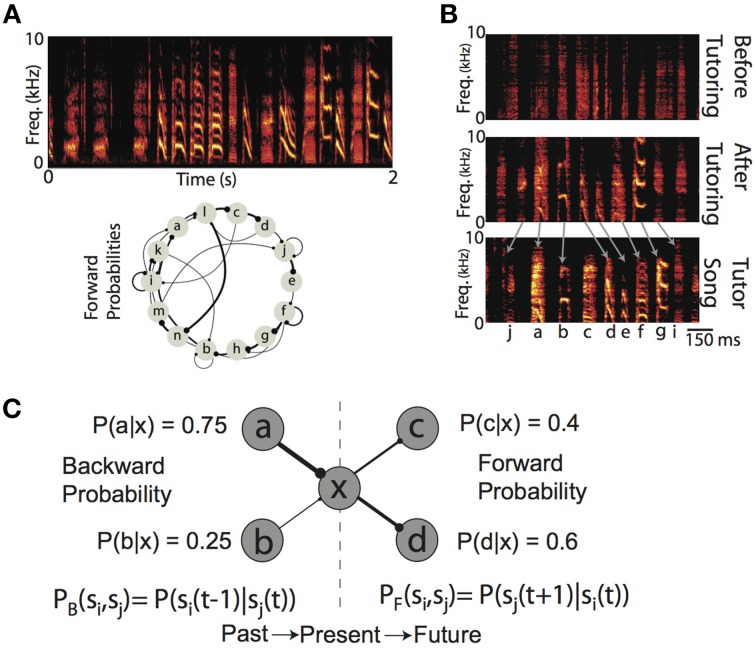
**Production and learning of sequences and their probabilistic characterizations. (A)** (top) Example spectrogram (power at frequency vs. time) of the song from one Bengalese finch. Songs are composed of categorical vocalizations (syllables), organized into probabilistic sequences. (bottom) Forward probability transition diagram for the song displayed above. Here, each node corresponds to a syllable, and edge width corresponds to conditional probability of transitioning to a syllable from a given syllable. Dots at the end of each edge denote the forward direction of song during production. **(B)** Example spectrograms of the song of one Bengalese finch recorded at a juvenile age (55 days of age, top) before exposure to tutor song, the song from the same bird after 10 days of tutoring, (65 days of age, middle), and the song of the tutor (bottom). To ease visual demonstration of learning, we have chosen a bird trained with a linear sequence of syllables. **(C)** Backward and forward probabilities have different semantics. For a given state of the system (e.g., “x”), the backward probability describes the distribution of previous states [P_B_(s_i_, s_j_) = P(s_i_(t−1)|s_j_(t))]. In contrast, the forward probability describes the distribution of upcoming states [P_F_(s_i_, s_j_) = P(s_j_(t+1)|s_i_(t))]. Generally speaking, for a given transition, the backward and forward probabilities will not be equal [e.g., P_B_(xc) = 1, P_F_(xc) = 0.4].

Many complex behaviors, ranging from the mating rituals of insects, to the vocalizations of humans and birds, unfold overtime not as a single, ballistic movement, but as coordinated sequences of movements (Lashley, 1951; Sternberg et al., 1978; Rhodes et al., 2004; Glaze and Troyer, 2006; Bouchard and Chang, 2014). The sensory world also exhibits temporal/sequential structure, and so perception can be thought of as processing a series of sequential events (Mauk and Buonomano, 2004; Yang and Shadlen, 2007; Bouchard and Brainard, 2013). A hallmark of many complex behaviors (as well as the sensory world) is that the sequencing of elements is, generally speaking, not stereotyped (i.e., not a linear sequence) but instead exhibits rich statistical structure that characterizes the probabilities with which one event transitions to/from any other event. For example, within a given language, a speech sound will transition to any other sound with some conditional probability, and this probability has perceptual and behavioral affects (Saffran et al., 1996; Peña et al., 2002; Vitevitch and Luce, 2005). Another example of a learned behavior (and sensory stimulus) exhibiting a rich probabilistic sequential structure is the song of the Bengalese finch, a songbird in which the probability of transitioning between distinct “syllables” is variable (Jin, 2009; Jin and Kozhevnikov, 2011; Katahira et al., 2011; Warren et al., 2012) (Figure 1A). In both humans and birds, vocal sequencing is not the result of a genetic program, and so must be learned (Figure 1B). The existence of these learned, probabilistically sequenced behaviors demonstrates that neural circuits are capable of learning to encode the transition probability between one elementary behavioral action and another. Furthermore, recent electrophysiological recordings from both humans and birds have shown modulations of neural activity that encode the conditional probability of sensory events (Bouchard and Brainard, 2013; Leonard et al., 2015). The ubiquity of such learned probabilistic sequences suggest that the learning mechanisms that engrain this probabilistic structure in neural circuits are just as general. However, these mechanisms are not fully understood. Our goal is to more fully understand the properties of local Hebbian plasticity rules that allow the development of synaptic weights directly proportional to the probabilistic structure of sequential activations imposed on neural networks.

In the nervous system, the biophysical substrate of learning and memory is instantiated, in part, by the strength of the synaptic connections between neurons (Dayan and Abbott, 2001; Koch, 2004). Furthermore, a large body of experimental work supports the notion that the modification of the synaptic weight between two neurons is dictated, in part, by the correlation between their activations (“neurons that fire together, wire together”) (Hebb, 1949; Dan and Poo, 2004). Prior theoretical studies of such “Hebbian-type” plasticity rules revealed that competition between synapses is required to shape synaptic weight distributions to reflect input/output correlations (Miller, 1996; Kempter et al., 2001; Sjöström et al., 2001; Gütig et al., 2003). Synaptic competition imbues Hebbian learning with the property that, as one set of synapses increases its weight, the other synapses decrease their weights (Miller et al., 1989; Miller, 1996; Song et al., 2000; Babadi and Abbott, 2010). The competitive Hebbian mechanisms typically used to model changes to synaptic weights will tend to drive those weights toward a binary distribution (min or max values), and in some situations this is the desired outcome (Amari, 1972; Abbott and Blum, 1996; Rubin et al., 2001; Song and Abbott, 2001; Gütig et al., 2003; Babadi and Abbott, 2010; Fiete et al., 2010). However, how this competition is tempered to engrain synaptic weights that span the entire range of values and represent the rich structure of probabilistic sequences is not fully understood.

Probabilistic sequences can be simultaneously characterized by the forward probabilities and the backward probabilities of transitions between states. Given the current state of a system, the forward probability [alternatively referred to as divergence probability (Bouchard and Brainard, 2013)] is the probability of transitioning to upcoming states from the present state [P_F_(s_i_,s_j_) = P(s_j_(t+1)|s_i_(t))]; it is the distribution of future events given the present (Figure 1C). Conversely, the backward probability [alternatively referred to as convergence probability (Bouchard and Brainard, 2013)] is the probability of transitioning from previous states to the present state [P_B_(s_i_,s_j_) = P(s_i_(t−1)|s_j_(t))]; it is the distribution of past events given the present (Figure 1C). Classical supervised synaptic learning rules (“outstar” and “instar” learning) suggest that learning these distinct probabilities is accomplished by modifying the outgoing (i.e., pre-synaptic) and incoming (i.e., post-synaptic) synaptic weights, respectively (Amari, 1972; Grossberg, 1986). More recent approaches have designed learning algorithms that either modify synaptic weights to directly minimize the difference between neuronal activations (Brea et al., 2013), or to match the probability that a stimulus leads to a reward (Soltani and Wang, 2010). In contrast to these supervised learning rules, recent modeling attempts to learn linear sequences with unsupervised timing-based Hebbian plasticity rules have suggested the importance for both pre- and post-synaptic competition in learning (Jun and Jin, 2007; Fiete et al., 2010), but have not disentangled their individual roles. Thus, the role of pre-synaptic and post-synaptic competition in learning of forward and backward probabilities has not been fully examined.

A specific motivating example comes from our recent electrophysiology results in nucleus HVC of Bengalese Finches (Bouchard and Brainard, 2013). Here, it was found that auditory responses of HVC neurons to a syllable increase in a monotonic, nearly linear fashion as a function of increasing backward probability of sequence production. Another motivating example comes from recent work in humans demonstrating that auditory neural responses in the superior temporal gyrus can be (linearly) positively modulated by both the forward and backward probabilities of speech sequences occurring in English (Leonard et al., 2015). Both of these results are at odds with theories of efficient or predictive sensory coding, which generally theorize that neural responses should decrease for more predictable stimuli (Barlow, 1961; Bastos et al., 2012). However, we note that for neurons operating in the linear regime of firing rates as a function of synaptic input, the observed linear increase in responses as a function of conditional probability implies a similar relationship between synaptic weights and conditional probabilities. We therefore hypothesized that Hebbian plasticity could mold synaptic weights to be directly proportional to either the forward or backward probabilities experienced by a network (Bouchard and Brainard, 2013; Leonard et al., 2015).

We focus on the specific problem of shaping the synaptic weights between two nodes in a network to reflect the probability of transitioning between those nodes. Throughout, sequential structure is imposed on the nodes of the network from external sources, but synaptic connectivity also contributes to network activity. This has been termed “learning with a teacher” in the literature (Legenstein et al., 2005). If we conceptualize the nodes as being neural elements in a motor network that are associated with distinct actions, then we can think of this problem as understanding how an experienced sequence of actions (and the corresponding sequence of activations of the nodes representing those actions) can shape the connections between nodes to engrain transition probabilities to produce those sequences (Jin, 2009). Alternatively, if we conceptualize the nodes as being elements of a sensory network that are associated with distinct events, then we can think of this problem as understanding how an experienced sequence of sensory events can shape the connections between nodes to engrain transition probabilities to recognize those sequences (Bastos et al., 2012). Therefore, our framework is equally applicable to the questions of how a network learns to produce probabilistic sequences or how a network learns to encode information about the statistics of variably sequenced sensory input.

Our results emphasize two key features of such probabilistic encoding by Hebbian plasticity. First, we show that either the forward or backward probabilities can be engrained in neural networks with unsupervised learning rules, depending on whether synaptic competition is exhibited pre-synaptically or post-synaptically. Second, we demonstrate that an appropriate balance between homogenizing forces (that tend to equalize all weights) and competitive forces (that tend to “binarize” the weights) can result in the stable development of weights that continuously span the range of transition probabilities, and that a small range of this balance can be optimal to encode a wide range of distributions.

## Methods

### Ethics statement

All procedures were performed in accordance with established animal care protocols approved by the University of California, San Francisco Institutional Animal Care and Use Committee (IACUC).

### Probabilities

The forward probability characterizes the frequency of transitioning from the current state (s_i_) to any other state s_j_ on the next time-step: P_F_(s_i_, s_j_) = P(s_j_(t+1)|s_i_(t)). The backward probability characterizes the frequency of transitioning to the current state (s_i_) from any other state s_j_ on the previous time-step: P_B_(s_i_, s_j_) = P(s_j_(t−1)|s_i_(t)).

### Recurrent network simulations

Our neural network simulations consisted of a two-layer network composed of an input layer that projected in a feed-forward manner to a recurrently connected layer. We conceptualize the input layer as corresponding to a sensory area and the recurrent layer as corresponding to a sensory-motor layer used for sequence generation, though this need not be the case. To simulate sequence learning in recurrent networks, one input unit is activated on each time step probabilistically based on the forward probabilities. A network of n linear-saturating firing rate based units (firing rates y_k_(t), k ∈ [1 n]) with Poisson background rates (η) are connected by recurrent weight matrix M (M_*t*=0_ ~ 1/n). On each time step, one unit is activated by a large input signal (δ(t)):

(1) $$\begin{array}{r} \text{y}\left(\text{t}\right)=\text{min}\left(\text{My}\left(\text{t}-1\right)+\text{δ}\left(\text{t}\right)+\text{η},{\text{r}}_{\text{max}}\right) \end{array}$$

Here, the min function imposes a hard, saturating non-linearity on the maximum firing rate (r_max_) of each unit. Thus, the firing rate of recurrent units is a linear combination of the current input units and recurrent units, up to r_max_.

All simulations of the recurrent neural networks began with a randomly seeded initial weight matrix (±5% deviation from 1/n). During each simulation, the network experienced a unique sequence of syllable transitions that obeyed the 1st-order Markov probabilities of a transition matrix, starting with a randomly selected syllable. All simulations were run for 1000 iterations of simulated “song” experience; here, one “song” was defined as a stochastic sequence of length 5 × n, n = number of states in the transition matrix. All results are averaged over five runs of the simulations that were initialized with different random weight matrices (M_*t*=0_ = (1/n)+ε) and experienced unique stochastic instantiations of the forward probabilities.

For simulations involving Gaussian transition matrices, because each state experienced nearly identical statistics and started from a random value from the same distribution, each state can be treated as an independent run. Thus, these simulations were run only once. Gaussian transition matrices consisted of 19-states with normally distributed forward probabilities between states, with the maximal transition probability (mean of Gaussian) from the ith state located at the i+9th state: $P\left(s_{j}|s_{i}\right)=\;N\left(i+9,\sigma^{2}\right).$

### Hebbian covariance rule

At each time step, the elements of the synaptic weight matrix M are changed according to a modified Hebbian covariance rule:

(2) $$\begin{array}{r} \Delta {\text{m}}_{\text{ij}}=\left\{ \begin{array}{c} {\text{A}}_{+}\sigma_{\text{i}}\sigma_{\text{j}}{\text{f}}_{+}({\text{m}}_{\text{ij}})\;\;\text{if }\sigma_{\text{i}}>0\vee \sigma_{\text{j}}>\;0\\ {\text{A}}_{-}\sigma_{\text{i}}\sigma_{\text{j}}{\text{f}}_{-}({\text{m}}_{\text{ij}})\;\text{if sign}(\sigma_{\text{i}})\neq \text{sign}(\sigma_{\text{j}}) \end{array}\right. \end{array}$$

Here, s_i_ is the firing rate deviation of pre-synaptic unit i at time t−1 (σ_i_ = *y*_*i*_(t−1)$-\bar{\text{y}i}$), σ_j_ is the firing rate deviation of post-synaptic unit j at time t (σ_j_ = y_j_(t−1)$-\bar{{\text{y}}_{\text{j}}}$), and A_±_ are the learning rates. In the simulations, the average ($\bar{\text{y}}$) is taken over the previous 5 time points. The value of 5 was arbitrarily chosen; similar results were obtained taking the average over the entire history. The functions f_±_(m_ij_) describes the magnitude of synaptic changes as a function of the current synaptic weight (see below). In this Hebbian covariance plasticity rule (HCP), a synapse between pre-synaptic unit i and post-synaptic unit j (m_ij_) is potentiated if and only if the firing rate of both the pre-synaptic (y_i_) and post-synaptic units (y_j_) are above their mean (σ_i_ > 0 ∨ σ_j_ > 0) while depression is induced if either pre- or post-synaptic firing rates is below its mean while the other is above the mean ([σ_i_ > 0 ∨ σ_i_ < 0] ∧ [σ_i_ < 0 ∨ σ_j_ > 0]). If both y_i_ and y_j_ are below their means, no change in synaptic weight occurs. Heterosynaptic competition was induced through the use of divisive normalization to maintain the sum of either pre- or post-synaptic weights at 1 (the denominator for presynaptic-competition: $\overset{N}{\underset{j=1}{\sum }}m_{.j}$; the denominator for post-synaptic-competition: $\overset{N}{\underset{i=1}{\sum }}m_{i.}$). Also, all weights were constrained to be positive (i.e., M ∈ [0, 1]) after every weight update by clipping. The exact schedule of enforcement of these constraints was found to not be crucial.

We use α to denote the relative magnitude of LTD (A_−_) to LTP (A_+_) and refer to it as the depression-potentiation ratio:

(3) $$\begin{array}{rc} \alpha =\frac{A_{-}}{A_{+}} & \end{array}$$

In the simulations, α is modulated by increasing the magnitude of depression while holding the magnitude of potentiation constant, and affects the amount of competition in Hebbian plasticity, which pushes weights toward their boundaries (Song et al., 2000). Here, we allowed α to range from [1 2] to stay within neurobiologically plausible values. In our simulations, the parameter β ∈ [0 1] controls the weight dependence of synaptic change via the power-law equations (from Gütig et al., 2003):

(4) $$\begin{array}{rcll} {\text{f}}_{+}\left(m_{ij}\right) & = & {\left(1-m_{ij}\right)}^{\beta } & \\ {\text{f}}_{-}\left(m_{ij}\right) & = & {{\text{m}}_{\text{ij}}}^{\beta } \end{array}$$

Gutig et al. have shown that β controls the amount of homogenization expressed by temporally asymmetric Hebbian plasticity, which pulls weights toward a single intermediate value.

### Quantification of simulation results

We performed a grid search over α (α ∈ [1:0.05:2]) and β (β ∈ [0:0.02:1]), and calculated the average absolute difference between the weight matrices and the conditional probabilities for each [α, β] pair. The accuracy of learning as a function of time was measured as the mean absolute difference (L_1_-norm) between the weight matrix and the appropriate transition matrix:

(5) $$\begin{array}{r} Err\left(t\right)=\frac{1}{N^{2}}\sum_{i,j=1}^{N}|m_{ij}\left(t\right)-P_{ij}| \end{array}$$

We did not use the Kullback-Leibler Divergence to measure learning accuracy because many entries in the transition matrices are 0, leading to division by zero in the denominator. A subset of simulations was run using L_2_-norm, and results did not qualitatively change.

Here we introduce the function Ψ(α, β) to describe the combined effect of α and β on learning:

(6) $$\begin{array}{r} \text{Ψ}(\text{α},\text{β})=\left\{ \begin{array}{c} \frac{2(\text{β}-0.5)}{\alpha };\beta \geq 0.5\\ \alpha (\beta -0.5);\beta <0.5 \end{array}\right. \end{array}$$

For the values of α and β examined here, Ψ(α, β) ranges from [−1 1], taking [α = 2, β = 0] to −1 and [α = 1, β = 1] to 1. Although not a theoretically derived quantity, the function Ψ is useful to simultaneously capture the effects of α and β on learning by quantifying the relative strengths of competitive and homogenizing forces expressed by HCP.

The average entropy of the weight matrix as a function of time was calculated as:

(7) $$\begin{array}{r} H\left(t\right)=\frac{1}{N}\sum_{i=1}^{N}\left(\sum_{j=1}^{N}m_{ij}\left(t\right){log}_{2}\left(m_{ij}\left(t\right)\right)\right) \end{array}$$

We additionally calculated the transition of entropy of single syllables as:

(8) $$\begin{array}{r} h\left(s_{i}\right)=\sum_{j=1}^{N}P\left(s_{j}|s_{i}\right)log_{2}\left(P\left(s_{j}|s_{i}\right)\right) \end{array}$$

The average final error and entropy surfaces as a function of the learning parameters α and β Err(α, β, *t* = 1000), H(α, β, *t* = 1000) resulting from the 16 unique transition matrices (derived from Bengalese finch songs) were smoothed by convolution with a 3 × 3 square of ones. All derivatives were computed as the slope from linear regression. All regressions for both linear and non-linear functions were done using built-in functions in Matlab.

### Spiking network simulations

Our spiking network consisted of a single pre-synaptic neuron contacting 17 post-synaptic neurons to examine learning of forward probability, and a single post-synaptic neuron with 17 pre-synaptic neurons to examine learning backward probability. All neurons had background Poisson spike rate of ~5 Hz. These neurons were driven by a “training signal” that injected current to produce spike trains in the network according to a predefined spike probability distribution conditioned on spiking in the presynaptic neuron. Specifically, for learning P_B_, “signal” spikes in the presynaptic neurons (x_j_) occurred every 20 ms (50 Hz mean rate) and preceded spikes in post-synaptic neuron (y) by 5 ms. The underlying conditional spike probability distribution P(y|x_j_) was a Gaussian centered on the 8th post-synaptic unit [*N*_(8, 2)_]. Time progressed in 1 ms intervals. We used conductance based leaky integrate-and-fire neurons with the following generic parameter values: V_th_ = −54 mV, V_reset_ = −60 mV, τ_m_ = 10 ms, E_leak_ = −70 mV, E_ex_ = 0 mV, E_in_ = −80 mV. The spiking activity of all neurons had an absolute refractory period of 5 ms (200 Hz max firing rate). All conductances had a decay constant of 2 ms. Synaptic conductances from the training signal to each neuron were large (g_signal_ = 2.5 mS/cm^2^), such that a spike was generated on every input pulse (as long as not within refractory period). We are interested in cases where the training signal dominates the dynamics of the network, and for simplicity synapses from the pre-synaptic neurons had small maximal conductance (g_ff_ = 0.1 mS/cm^2^), which generally did not cause spiking activity. Generally speaking, we require that the training signal dominates the correlated spiking in the network, and deviations from this will likely interfere with learning of the training signal. Learning was modestly affected by increasing the background Poisson spiking rate, mostly resulting in a slower rate of learning.

### STDP

According to the STDP rule (Song et al., 2000; Kempter et al., 2001; Gütig et al., 2003), the change in synaptic strength between a pre-synaptic and post-synaptic neuron (Δm_ij_) decays exponentially (with decay rates τ_±_ = 10 ms) as the temporal difference in their spike times (Δt) increases:

(9) $$\begin{array}{r} \Delta {\text{m}}_{\text{ij}}\left(\Delta \text{t}\right)=\left\{ \begin{array}{r} {\text{A}}_{+}\text{exp}\left(\Delta \text{t}∕\tau_{+}\right){\text{f}}_{+}\left({\text{m}}_{\text{ij}}\right)\Delta \text{t}<0\\ {\text{A}}_{-}\text{exp}\left(-\Delta \text{t}∕\tau_{+}\right){\text{f}}_{-}\left({\text{m}}_{\text{ij}}\right)\Delta \text{t}\geq 0 \end{array}\right. \end{array}$$

A_+_ = 0.001 and A_−_ = αA_+_, describe the magnitude of synaptic change. f_±_(m_ij_) are functions that describe the relative magnitudes of synaptic potentiation and depression differ and how the magnitude of synaptic change depends on the current synaptic weight.

### Song collection and analysis

Sixteen adult male Bengalese finches (age > 110 days) were used in this study. During the experiments, birds were housed individually in sound-attenuating chambers (Acoustic Systems, Austin, TX), and food and water were provided *ad libitum*. 14:10 light:dark photo-cycles were maintained during development and throughout all experiments. Birds were raised with a single tutor. All behavioral analyses were done using custom code written in Matlab (The Mathworks, Natick, MA). An automated triggering procedure was used to record and digitize (44,100 Hz) several hours of the bird's singing. These recordings were then scanned to ensure that more than 50 bouts were obtained. Bouts were defined as continuous periods of singing separated by at least 2 s of silence. Bengalese finch songs typically consist of between 5 and 12 distinct acoustic events, termed syllables, organized into probabilistic sequences. Each bout of singing consists of several renditions of sequences, with each sequence containing between 1 and approximately 40 examples of a particular syllable. The syllables from 15 to 50 bouts were hand labeled. These transition matrices for the 16 birds used here are representative of the structure of Bfs songs, and exhibit diversity in the number of syllables (range = [5−10]) and degree of sequence entropy (range = [0.1−2.8] bits).

## Results

### The site of synaptic competition dictates the temporal flow of information engrained by correlation based Hebbian learning

**Figure 2:**
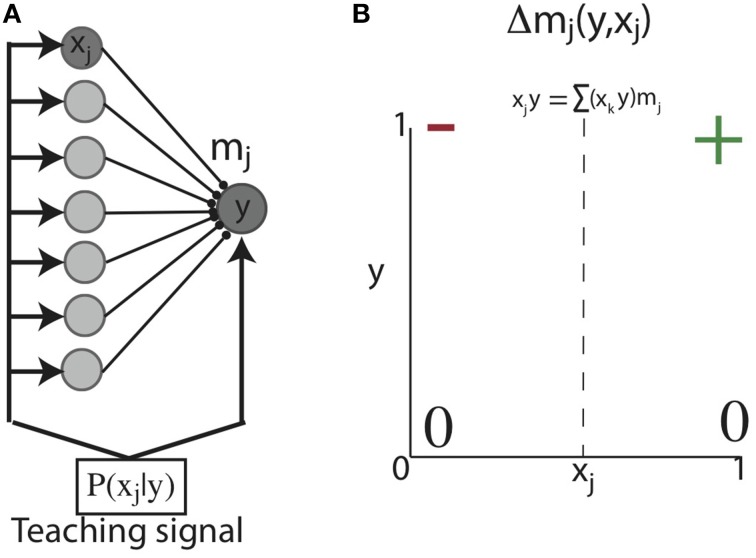
**Learning conditional probabilities with correlation based Hebbian plasticity in binary networks. (A)** Feed-forward network architecture and inputs. A single post-synaptic unit with binary activation (y) receives feed-forward synaptic contacts from a population of binary pre-synaptic units (x_j_). This network is activated such that at each point in time, one pre-synaptic unit (x_j_) is co-activated with the post-synaptic unit (y) (e.g., dark gray nodes). **(B)** Hebbian correlation learning rule for networks with binary activation units. A synapse between x and y can change only if the post-synaptic neuron fires (*y* = 1). Then if the correlation between x and y exceeds the total correlation between y and all the other synapses, the synapse is potentiated (+); otherwise the synapse is depressed (−).

We initially considered a feed-forward network of a single post-synaptic unit (y) connected to pre-synaptic units (x_j_) through weights m_j_. This network is activated by an input sequence such that during some plasticity window (Δt), x_j_ and y are sequentially activated with probability P_B_ = P(x_j_ = 1|y = 1) (Figure 2A) (below, for notational simplicity, we use e.g., “y” for “y = 1”). With such a network, our first objective was to show analytically that Hebbian plasticity can shape initially unstructured weights to reflect P_B_ under simplified conditions. Therefore, for analytic tractability, we considered the situation in which the units of this network exhibit binary activations (i.e., 0 or 1), which can be loosely associated with whether or not a neuron discharged an action potential. With such binary activations, correlation based Hebbian plasticity dictates: a synapse between x and y changes only if the post-synaptic neuron fires (y = 1). Then if the correlation between x and y exceeds the total correlation between y and all the other synapses, the synapse is potentiated, otherwise the synapse is depressed (Figure 2B). We aim to show that Hebbian plasticity with post-synaptic competition can develop synaptic weights equal to the probability that the pre-synaptic unit is active with a post-synaptic unit, conditioned on the activity of the post-synaptic unit; that is, the backward conditional probability:

(i) $$\begin{array}{r} m_{j}\to P\left(x_{j}|y\right) \end{array}$$

Under a correlation based Hebbian learning rule with weight decay based on the sum of the pre-synaptic weights, synapses change according to the equation:

(ii) $$\begin{array}{r} {△m}_{j}\propto x_{j}y-\sum_{k=1}^{N}\left(x_{k}y\right)m_{j} \end{array}$$

Thus the weight change is proportional to the difference of two terms, where the constant of proportionality is a fixed, small learning rate. The first term on the right-hand side is the correlation between post-synaptic unit (y) and the pre-synaptic unit (x_j_) (i.e., the Hebbian term), and the second term imposes a leak, or decay on the post-synaptic weights whose coefficient is equal to the summed correlation between inputs and the output, which induces heterosynaptic competition. For a small enough learning rate, the weight dynamics in Equation (ii) temporally integrates the statistics of pre and post-synaptic activity and approaches, on average, a steady state determined by the equation:

(iii) $$\begin{array}{r} 0=\left\langle x_{j}y\right\rangle -\sum_{k=1}^{N}\left\langle x_{k}y\right\rangle m_{j} \end{array}$$

Because both and x and y are binary, the expected value of the correlation between x_j_ and y (<x_j_y>) is equal to P(y|x_j_)P(x_j_). Therefore we can re-write (iii) as:

(iv) $$\begin{array}{r} 0=P\left(y|x_{j}\right)P\left(x_{j}\right)-\sum_{k=1}^{N}P\left(y|x_{k}\right)P\left(x_{k}\right)m_{j} \end{array}$$

Solving for m_j_ gives:

(v) $$\begin{array}{r} m_{j}=\frac{P\left(y|x_{j}\right)P\left(x_{j}\right)}{\sum_{k=1}^{N}P\left(y|x_{k}\right)P\left(x_{k}\right)} \end{array}$$

The denominator simplifies via marginalization over *x*_*k*_ to *P*(*y*), yielding:

(vi) $$\begin{array}{r} m_{j}=\frac{P\left(y|x_{j}\right)P\left(x_{j}\right)}{P\left(y\right)} \end{array}$$

which, by Bayes' Rule, gives:

(vii) $$\begin{array}{r} m_{j}=P\left(x_{j}|y\right) \end{array}$$

The proof for learning forward probability with pre-synaptic Hebbian plasticity in networks of a single pre-synaptic unit (x) connected to multiple post-synaptic units (y_i_) follows by appropriate replacement of variables: if ${△m}_{i}=\;y_{i}x-\;\sum_{k=1}^{N}\left(y_{k}x\right)m_{i}$, then *m*_*i*_ → *P*(*y*_*i*_|*x*).

Together, these results demonstrate that Hebbian plasticity with a post-synaptic locus of competition can develop weights equal to backward conditional probabilities, while a pre-synaptic locus of competition can develop weights equal to the forward conditional probabilities. We note that for the very simple case of binary neural activations, we could have simply replaced the coefficient of the leak term in Equation (ii) with the post-synaptic activity y, and the entire proof for learning conditional probabilities would go through. We chose the more complex scheme to maintain a level of parallelism with the more biologically realistic rules below that can learn sequential statistical structure without the need for high SNR binary neural activations.

### Pre-synaptic and post-synaptic competition in Hebbian covariance plasticity engrain forward and backward conditional probabilities

With the intuition gained above, we next investigated the Hebbian mechanisms that engrain conditional probabilities in the synaptic weights of fully recurrent networks of rate-based units. For these networks, each node is initially connected to each other node in the network. As before, each node can be associated with a neural element that represents a discrete motor action or sensory event, and each element is activated by a single external input (“teaching” signal). The goal of learning, then, is to engrain the transition probabilities between events in the synaptic weights connecting nodes in the network. As we will describe, the proposed network properties and learning rule provide an intermediate framework between the analytically tractable binary network trained with correlation based Hebbian plasticity and more biophysically realistic spiking network trained with STDP examined at the end of the paper.

**Figure 3:**
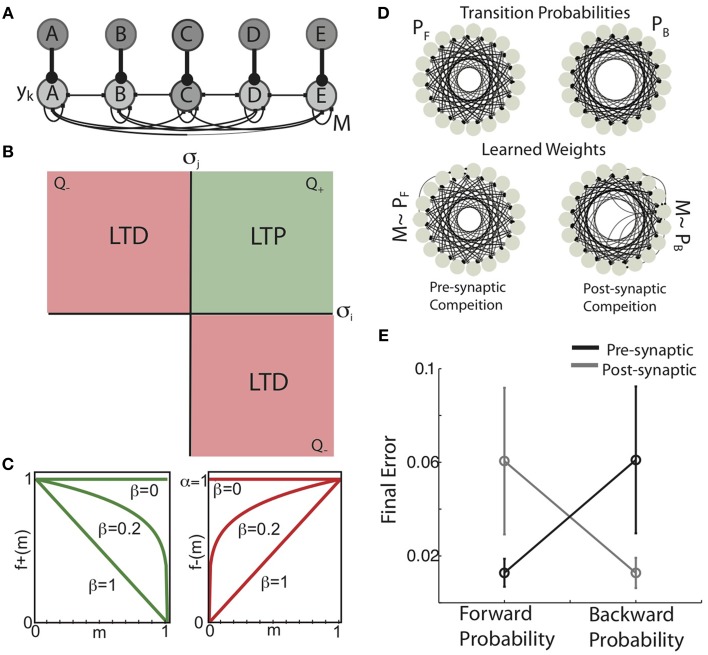
**Pre-synaptic and post-synaptic competition in Hebbian covariance plasticity engrain forward and backward conditional probabilities. (A)** Recurrent network architecture and inputs. Rate-based units (y, light-gray nodes) were recurrently connected by the excitatory weight matrix M. Each y corresponds to a unique sequence element, and receives strong “sensory” input (dark-gray nodes) corresponding to the presentation of that element in the sequence. The activity of recurrent units is a linear up to a hard-threshold to ensure stable network dynamics, and is a combination of recurrent inputs from excitatory synaptic connectivity and the sequential activity imposed by the inputs. **(B)** Hebbian covariance plasticity rule. A synapse between pre-synaptic unit i and post-synaptic unit j (m_ij_) is potentiated if the firing rate of both the pre-synaptic (y_i_) and post-synaptic units (y_j_) is above there mean (positive variances, green quadrant, Q_+_), while depression is induced if either pre- or post-synaptic firing rate is below mean its mean while the other is above the mean (negative covariance, red quadrants, Q_−_). If both y_i_ and y_j_ are below their means, no change in synaptic weight occurs (blank quadrant). **(C)** Weight dependence of synaptic change. The weight dependence of synaptic potentiation [f_+_(m), green] and synaptic depression [f_−_(m), red] for three values of the parameter β. When β = 0, the magnitude of potentiation and depression are independent of the synaptic weight (“additive” HCP). In contrast, when β = 1, the magnitude of potentiation decreases linearly with increasing synaptic weight, while the magnitude of depression increases linearly (“multiplicative” HCP). **(D)** (top) Example forward transition diagram (left) used as input to the recurrent network, and the corresponding backward transition diagram (right). Here, as in Figure 1B, each node in the diagram corresponds to a unique element of the sequence and the edges connecting nodes are proportional to the transition probabilities connecting those nodes. (bottom) Synaptic connectivity displayed as network graphs resulting from pre-synaptic competition (left) post-synaptic competition (right). Here, each node in the diagram corresponds to a network element and the edges connecting nodes are proportional to the synaptic weight connecting those nodes. Focusing on the global structure of these graphs, it is apparent that presynaptic competition takes the weight matrix to the forward probability distribution (M ~ P_F_), while post-synaptic competition takes the weight matrix to the backward probability distribution (M ~ P_B_). **(E)** Mean absolute error (mean ± s.d., *N* = 8) between the learned weight matrices (M) and the forward and backward probability matrices when either pre-synaptic competition (black) or post-synaptic competition (gray) was instantiated in HCP.

A network of linear-saturating firing rate based units (firing rates y_k_(t), k ∈ [1 n]) with Poisson background rates (η) are connected by recurrent weight matrix M (M_t=0_ ~ 1/n) (Figure 3A). On each time step, one unit is activated by a large input signal (δ(t)) (Equation 1). The distribution of δ(t) determines the probabilistic sequence experienced by the network and is completely determined by the forward transition probabilities, P_F_(s_i_,s_j_) and the (randomly chosen) initial state. In this network, the activity of recurrent units is a function of both the imposed sequence and the recurrent connectivity matrix.

Hebbian plasticity was applied to M based on the firing rate vectors at consecutive times (y(t−1), y(t)). Here, we implemented a biologically plausible, Hebbian covariance learning algorithm (Figure 3B). In Hebbian covariance plasticity (HCP), a synapse between pre-synaptic unit i and post-synaptic unit j (m_ij_) is potentiated if and only if the firing rate of both the pre-synaptic (y_i_) and post-synaptic units (y_j_) is above their mean (green quadrant, Q_+_), while depression is induced if either pre- or post-synaptic firing rates is below its mean while the other is above the mean (negative covariance, red quadrants, Q_−_). If both y_i_ and y_j_ are below their means, no change in synaptic weight occurs (blank quadrant) (Equation 2). The HCP learning rule reflects the intuition that changes in synaptic weight should be sensitive to the co-variation of pre- and post-synaptic firing rates. Furthermore, incorporating both pre and post-synaptic “deviations” makes HCP insensitive to changes in the mean firing rate, as is the case for STDP (Song et al., 2000; Kempter et al., 2001). Finally dictating that no plasticity occurs when both pre and post-synaptic firing rates are below their mean removes the un-biological potentiation of synapses when both neurons have negative deviations, and hence a positive covariance (Miller, 1996). It is noteworthy that the geometry of synaptic change in Hebbian covariance plasticity is similar to the correlation based learning rule for sequentially active binary units used for the analytic calculations (compare Figure 2B and Figure 3B).

Two major issues with Hebbian plasticity rules are competition and stability (Miller and MacKay, 1994; Miller, 1996; Song et al., 2000; van Rossum et al., 2000; Kempter et al., 2001; Rubin et al., 2001; Kepecs et al., 2002; Gütig et al., 2003; Babadi and Abbott, 2010). The relative magnitudes of LTD and LTP expressed by temporally asymmetric Hebbian plasticity (STDP) have been shown to affect the competitive force expressed during learning (Song et al., 2000; Kempter et al., 2001; Rubin et al., 2001; Gütig et al., 2003). Increasing the competitive force expressed by STDP pushes synaptic weights toward binary distribution (∀m_ij_ = [m_min_ ∧ m_max_]). We use α to denote the relative magnitude of LTD (A_−_) to LTP (A_+_) and refer to it as the depression-potentiation ratio (Equation 3). When α = 1 (Figure 3C), the maximal incremental amount of LTP and LTD are matched; when α = 2, the magnitude of LTD is twice as strong as LTP.

The weight dependence of synaptic change expressed by temporally asymmetric Hebbian plasticity has been shown to have dramatic affects on the steady state synaptic weight distributions induced by the learning process (van Rossum et al., 2000; Rubin et al., 2001; Kepecs et al., 2002; Gütig et al., 2003). The weight dependence of synaptic change influences the homogenizing force expressed by STDP, which pulls the synaptic weights toward a uniform distribution (∀m = 1/n, n = number of distinct inputs). In our simulations, the parameter β controls the weight dependence of synaptic change via the power-law equations (Figure 3C, from Gütig et al., 2003) (Equation 4). When β = 0, these equations dictate that the amount of LTP and LTD are independent of the current synaptic weight (“additive” rules) (Abbott and Blum, 1996; Song et al., 2000; Kempter et al., 2001; Rubin et al., 2001; Gütig et al., 2003). Conversely, when β = 1, the magnitude of LTP decreases linearly with increasing synaptic weight and the magnitude of LTD increases linearly with synaptic weight (“multiplicative” rules) (Kistler and van Hemmen, 2000; van Rossum et al., 2000; Rubin et al., 2001; Kepecs et al., 2002; Gütig et al., 2003). Varying β between these extreme values gives weight dependencies that smoothly interpolate between the additive and multiplicative STDP conditions (Gütig et al., 2003).

The emergence of synaptic weights that reflect the statistics of pre and post-synaptic firing patterns requires competitive mechanisms that dictate that as some synaptic weights increase, the other synaptic weights decrease (Miller and MacKay, 1994; Miller, 1996; van Rossum et al., 2000; Gütig et al., 2003). However, the most naïve formulation of Hebbian plasticity, even if mechanisms for depression are included, lacks the competitive force necessary to mold an initially random and statistically homogenous initial weight matrix into one that reflects the statistical structure of inputs and outputs (Miller and MacKay, 1994; Miller, 1996). Because of the positive feedback character of Hebbian plasticity (strong synapses lead to higher correlations between pre and post-synaptic activity, leading to potentiation, yielding stronger synapses, resulting in more potentiation), synaptic potentiation tends to grow to unrealistic values unless constrained in some way (Miller and MacKay, 1994; Miller, 1996; Song et al., 2000). Here, this positive feedback would be mediated by the recurrent synaptic weight matrix, M (Equation 1). In the correlation-based rule used for analytic calculations, competition is generated by the weight decay term in the update rule, while in STDP, competition is generated by having the magnitude of depression slightly larger than potentiation (i.e., α > 1). For rate-based Hebbian plasticity, it has been shown that divisively constraining the sum of synaptic weights solves both these problems (Miller and MacKay, 1994; Miller, 1996), and thus we incorporate divisive normalization in HCP. Normalizing the sum of synaptic weights to 1 also affords the interpretation of synaptic weights as probability distributions, enabling direct comparison of weights to transition probabilities. In this way, the goal of learning is to develop synaptic weights that are equal to the transition probabilities present in the input sequence.

We confirmed that HCP (with optimized parameters) instantiating pre-synaptic competition resulted in synaptic weight matrices that reflect the forward probability of the experienced sequences while post-synaptic competition developed weights that reflect the backward probabilities. For these simulations, the input sequence consisted of 19 states, each state having the same Gaussian forward conditional probabilities (P_F_(s_i_, s_j_) = N(i+9, σ^2^); s_i_, s_j_ ∈ [1 19]). An example of the difference in learned synaptic weights developed by pre vs. post-synaptic competition in HCP is displayed in Figure 3D. The forward transition diagram for this sequence is presented in the top-left and the corresponding backward transition diagram is on the top-right. The learned weight distributions presented in the bottom row (shown as network graphs), suggest that HCP with pre-synaptic competition developed recurrent weight matrices that reflect the forward probabilities of the experienced sequence (M ~ P_F_, bottom-left) while post-synaptic competition developed recurrent weight matrices that reflected the backward probabilities of the same sequence (M ~ P_B_, bottom-right). This is quantified for a variety of Gaussian transition matrices with varying σ^2^ in Figure 3E (data presented as mean ± s.d. across *N* = 8 different transition matrices, pre-synaptic competition black, post-synaptic competition gray). Together with the results of the previous section, these results demonstrate that the site of synaptic competition dictates whether the forward or backward probabilities of an input sequence are engrained in synaptic weights of neural networks.

### Hebbian encoding of Bengalese finch song sequences: an example

The above results demonstrate that Hebbian learning rules can shape synaptic weights to reflect conditional probabilities. However, these rules required a “balance” of the weight dependence of synaptic change and the depression-potentiation ratio. To more fully understand the Hebbian mechanisms that enable learning of biologically relevant, probabilistic sequences, we trained rate-based recurrent networks to encode the song sequences of Bengalese finch songs. Using the forward transition matrices derived from 16 Bengalese finch songs as our data set, we investigated how probabilistic encoding depends on the magnitude of competitive and homogenizing forces expressed by asymmetric Hebbian covariance plasticity. The network architecture and learning rules are the same as described in the previous section (Figures 3A–C), except that the number of nodes in the network varied according to the number of syllables in a given Bengalese finches' song.

**Figure 4:**
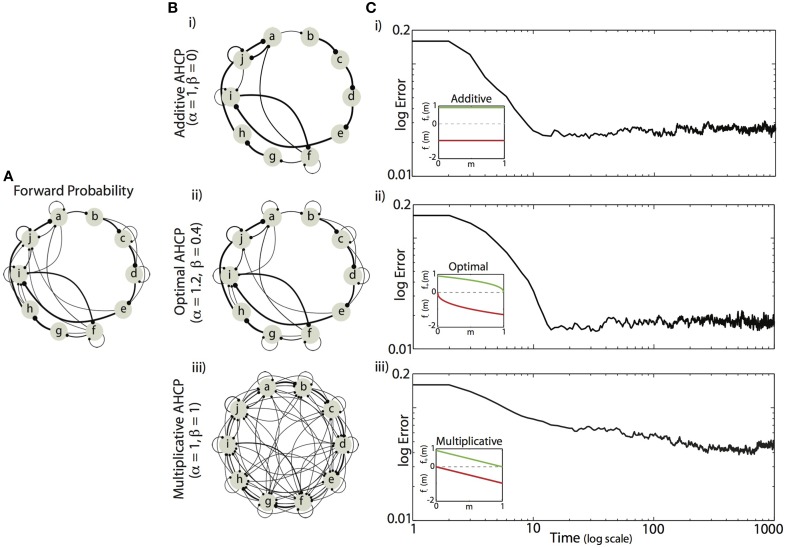
**Example of Hebbian encoding of Bengalese finch song sequences. (A)** The forward probabilities displayed in this diagram, derived from one Bf (same as Figure 1B), served as input to the recurrent network **(A)**. **(B,C)** Final synaptic connectivity (**B**, same format as in **C**) and learning time courses **(C)** for different magnitudes of competitive (α) and homogenizing forces (β). (i) Additive HCP (α = 1, β = 0). (ii) Optimal HCP (α = 1.2, β = 0.4). (iii) Multiplicative HCP (α = 1, β = 1). Insets in **(C)** display the corresponding weight dependence of synaptic change (green, LTP; red, LTD).

We found that minimizing the mean difference between the experienced forward probabilities and the learned synaptic weights (Equation 5) required a balance between the magnitude of competitive force (α) and the magnitude of homogenizing force (β). Example results from simulations using the forward probabilities in Figure 4A are displayed in Figures 4B,C, where final synaptic weight matrices are displayed as network graphs (Figures 4Bi–iii) and learning curves (Figures 4Ci–iii) are shown for three regimes of HCP. Here, we compare results from the optimal balance of α and β (α = 1.2, β = 0.4; b,c. ii) to results obtained from regimes that approximately correspond to additive HCP (α = 1, β = 0; b,c. i) and multiplicative HCP (α = 1, β = 1; b,c. iii). As can be seen in Figure 4Bi, additive HCP (β = 0) results in a final weight matrix (M_t=1000_) that over-strengthened the most probable transitions at the expense of the less probable transitions. This regime resulted in learning dynamics that were rapid: the majority of error reduction occurred within the first 10 “bouts” (one bout corresponds to 50 syllable presentations) (Figure 4Ci). In contrast, we found that multiplicative HCP (α = 1, β = 1) resulted in a final weight matrix that was a poor approximation of the experienced forward probabilities (Figure 4Biii). In further contrast to the additive condition, multiplicative HCP resulted in learning that was much slower (Figure 4Ciii). Finally, HCP with optimal values of α and β (α = 1.2, β = 0.4, inset in Figure 4Cii) resulted in a final weight matrix that was a good approximation of the experienced forward probabilities (Figure 4Bii). Here, we see that balancing the magnitudes of competitive and homogenizing forces allowed for synaptic weights that span the full dynamic range [0, 1], and still retained values intermediate between the extremes. Furthermore, the initial rate of learning was intermediate between the additive and multiplicative conditions. These results suggest that learning neurobiologically relevant probability distributions requires a balance of competitive and homogenizing forces, and that these forces affect both the final synaptic weight distribution and the rate at which structure emerges in those weights.

### The balance of competitive and homogenizing forces strongly influences the accuracy of probabilistic encoding

**Figure 5:**
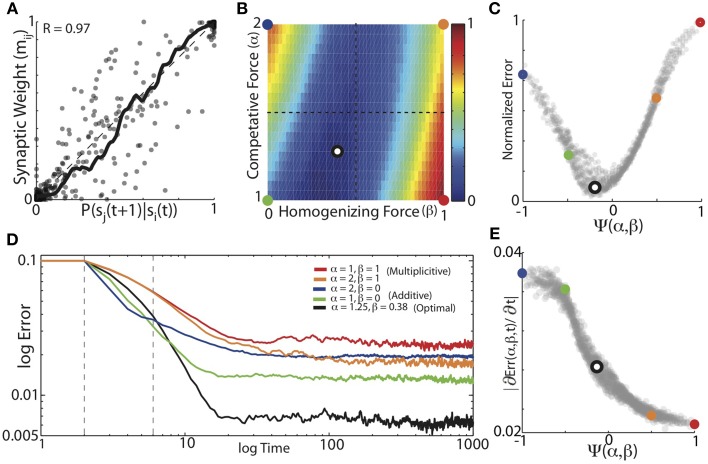
**The balance of competitive and homogenizing forces strongly influences the accuracy of probabilistic encoding. (A)** Conditional forward probabilities vs. learned synaptic weights resulting from HCP with optimal values of α and β. Here, each point corresponds to one transition/weight pairing (*n* = 1018 transitions). Thick black line is the locally smoothed mean, dashed line is unity. **(B)** Average normalized error surface as a function of the homogenizing forces (β) and the competitive forces (α) expressed by HCP. Colored dots demarcate the corners of the parameter space, and the black/white dot is the mean of the individual optimal values across the 16 different Bf transition matrices [α = 1.25, β = 0.38]. **(C)** Average normalized error as a function of the homogenization-competition ratio Ψ(α, β) (see text). Colored circles map to colored circles in **(B)**. Here, each dot corresponds to a pixel in **(B)**. **(D)** Average error across time. Five different regimes resulting from varying magnitudes of homogenizing and competitive forces are shown: “additive” (green: α = 1, β = 0); average optimal values (black: α = 1.25, β = 0.38), “multiplicative” (red: α = 1, β = 1); (orange: α = 2, β = 1); and (blue: α = 2, β = 0). Dashed gray vertical lines demarcate time period of slope measurements. **(E)** Magnitude of initial error reduction (∂Err(Ψ,t)/∂t) generally decreased as a function of increasing the magnitude of the homogenizing force.

We found that balanced HCP was able to mold initially unstructured synaptic weights to reflect the transition probabilities of Bengalese finch songs with high fidelity. We performed a grid search over α (α ∈ [1:0.05:2]) and β (β ∈ [0:0.02:1]), and calculated the average absolute difference between the weight matrices and the forward conditional probabilities for each α, β pair (Equation 5). The results for the optimal weight dependence of synaptic change (β) and the depression-potentiation ratio (α) for each of the 16 unique Bf songs used in our simulations are displayed in Figure 5A. Here, we plot the experienced forward probabilities for each transition vs. the learned synaptic weights corresponding to that transition (*n* = 1018, thick black line is local mean). As suggested by the examples in Figure 4, optimizing α and β resulted in synaptic weights that where highly correlated with the experienced forward probabilities of the input sequences (*R* = 0.97). Although there is a strong correlation between the final synaptic weights and the experienced forward probabilities, there is a fair amount of spread about the line of unity (which corresponds to a perfect encoding of forward probability in synaptic weights), an issue we return to later.

We systematically examined how the magnitudes of competitive and homogenizing forces affect the ability of HCP to engrain the experienced forward probabilities by parametrically varying α and β across the range α ∈ [1 2] and β ∈ [0 1]. The plot in Figure 5B displays the average normalized error surface as a function of α and β, which shows that optimal learning required a balance of the magnitudes of the weight dependence of synaptic change and the depression-potentiation ratio (mean optimal values are α = 1.25, β = 0.38, black dot with white interior). Furthermore, we observed that the lower-left region of the error surface generally had the lowest errors, suggesting that, for the transition matrices used here, relatively lower values of α and β result in more accurate learning. The error exhibits a smooth concave structure as the magnitude of the weight dependence of synaptic change increases, with a broad trough for intermediate values of β (mean width at 10% max = 0.3, i.e., 30% of β range). Both the width and minimum of the error surface exhibited linear dependencies on the depression-potentiation ratio: the width decreased (Δ = −0.14, *R*^2^ = 0.96 from linear regression) and the minimum shifted to the right as α increased (Δ = 0.27, *R*^2^ = 0.93 from linear regression). The error surface is relatively flat around the mean optimal values (black/white dot), suggesting that a single [α, β] pair could have been used for all input matrices with little effect on the final results.

For intermediate values of β (β~ 0.5), the error surface exhibited a negligible dependence on the magnitude of α. In contrast, for β values approaching the extremes, the error surface exhibited an approximately anti-symmetric dependence upon the depression-potentiation ratio. That is, when HCP expresses a weak homogenizing force (small β), increasing the competitive force (α) results in increased error. Conversely, when HCP exhibits strong homogenizing force (large β), increasing the competitive force (α) results in decreased error. The approximate anti-symmetry of the error surface described above suggests that a single, piece-wise defined function can capture the net affect of α and β on learning error. Here we use the function Ψ(α, β) to describe the combined effect of α and β on learning (Equation 6). For the values of α and β examined here, Ψ(α, β) ranges from [−1, 1], taking [α = 2, β = 0] to −1 and [α = 1, β = 1] to 1. In Figure 5C, we remap the error surface in Figure 5B using Ψ, which exhibits a well-defined global minimum (here, each point corresponds to one pixel in Figure 5B and colored points in Figure 5C correspond to colored points in Figure 5B).

We further sought to understand how the balance of competitive and homogenizing forces affected the dynamics of the learning process. In Figure 5D we present the average error trajectories for the five regimes demarcated in Figures 5B,C. As suggested by the examples in Figure 4, we found that regimes with the strongest competitive tone (blue, α = 2, β = 0 and green, α = 1, β = 0 curves), resulted in the most rapid initial learning while regimes with the strongest homogenizing tone (red, α = 1, β = 1 and orange, α = 2, β = 1) resulted in the slowest initial learning. We quantified the rate of learning by measuring the initial linear slope of the learning curves (between dashed vertical lines, Figure 5D) and plot the magnitude of these slopes as a function of Ψ in Figure 5E. We found that the magnitude of initial error reduction decreased nearly monotonically as the homogenizing tone increased (i.e., as Ψ went from −1 to 1). We observed that error time-courses for individual Bf transition matrices could exhibit an initial minimum in error followed by a slow increase to asymptote thereafter. However, the learning dynamics across the different transition matrices used here were heterogeneous, and so this aspect was washed out in the mean.

### Entropy of the learned synaptic weight distribution depends monotonically on the balance of competitive and homogenizing forces

Visual examination of the networks graphs in Figures 4Bi–iii suggested that, for a given target matrix, the balance of competitive and homogenizing forces expressed by HCP effects the variability of the synaptic weight matrices: as the magnitude of the homogenizing force increased, the variability of the steady-state weight matrix increased. We quantified the variability of synaptic weight matrices by calculating the average entropy, where the average is taken over states (e.g., syllables) (Equation 7). We systematically examined how the balance of competitive and homogenizing forces expressed by HCP affect the steady-state entropy of synaptic weight matrices. Unlike learning error, which describes the synaptic weights relative to the input (i.e., target) transition probabilities, calculation of entropy makes no reference to an external model. Entropy thus provides a measure of the intrinsic structure of the synaptic weight distributions.

**Figure 6:**
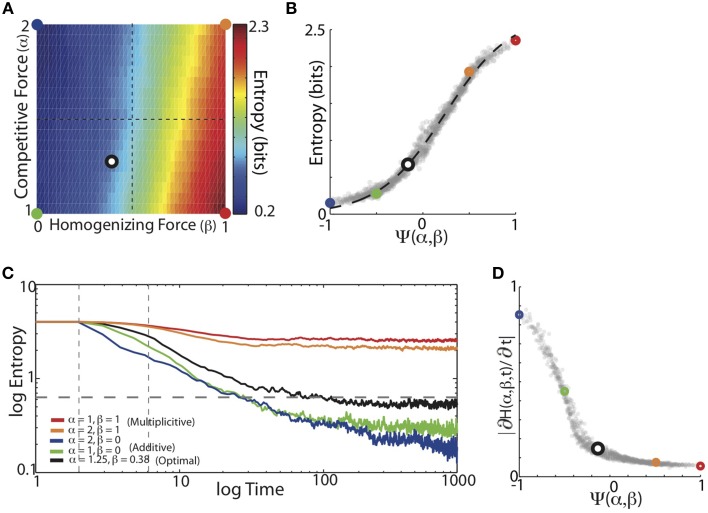
**Entropy of the learned synaptic weight distribution depends monotonically on the balance of competitive and homogenizing forces. (A)** Mean entropy of final synaptic weight distributions as a function of the homogenizing forces (β) and the competitive forces (α) expressed by HCP. Colored dots demarcate corners of parameter space and black/white dot corresponds is the mean of the individual optimal values across the 16 different Bf transition matrices [α = 1.25, β = 0.38]. **(B)** Average final entropy of the synaptic weight matrices as a function of Ψ. Colored points correspond to colored points demarcated in **(A)**. Black dashed line is best fitting hyperbolic tangent, which explains 99% of the variability (*R*^2^ = 0.99) in final entropy as a function of Ψ. **(C)** Mean entropy of synaptic weight distributions across time. Five different regimes from varying magnitudes of homogenizing and competitive forces are shown (colors same as in **A**). Gray horizontal dashed line is average entropy across the input matrices (0.62 bits). Gray vertical lines demarcate times at which slopes were measured. **(D)** Magnitude of initial entropy reduction (∂H(Ψ,t)/∂t) decreased as a function of increasing Ψ.

We found that the balance of competitive and homogenizing forces expressed by HCP monotonically modulated the entropy of the final synaptic weight matrix. Figure 6A displays the average entropy of the steady-state synaptic weight matrices resulting from HCP learning of the 16 Bf song sequence probabilities for varying α and β. Here we see that increasing β resulted in a corresponding increase in the entropy of the synaptic weights. In contrast to β, increases in α resulted in decreased entropy of the synaptic weight distributions. Additionally, changes in β resulted in larger changes to entropy then did changes in α: that is, changing β for a given value of α had a larger effect on entropy than changing α for a given value of β. This suggests that the magnitude of homogenization had a larger influence on final entropy than the magnitude of competition. We summarized how the balance of competitive and homogenizing forces effected the entropy of the steady-state synaptic weight distributions using the function Ψ(α, β). The plot in Figure 6B shows that the entropy of the synaptic weights monotonically increased as the value of Ψ went from −1 to 1. Here, the colored points correspond to the regimes demarcated in Figure 6A, and the dashed black line displays the best-fitting hyperbolic tangent function (*R*^2^ = 0.99). Thus, entropy increased with increased homogenization (van Rossum et al., 2000; Gütig et al., 2003).

We next examined the time-course of the entropy of synaptic weight distributions over learning. In Figure 6C we plot the average entropy trajectories for the same five conditions of α and β (colors), and we see that as time progressed, the entropy of the weight matrices steadily declined to asymptote. HCP with a stronger homogenizing tone (red and orange) gave rise to slower entropy reduction dynamics than HCP with stronger competitive tones (blue and green), while the optimal balance (minimum error, black) was again intermediate between these extremes. In contrast to the error trajectories, the rank ordering of entropy reduction was generally maintained across time (Figure 6C). The rate of entropy reduction (quantified as linear slope between dashed vertical lines in Figure 6C) was a monotonically decaying function of the magnitude of homogenization relative to competition (Figure 6D). The final entropy of the minimum error-learning rule (black) was slightly less than the average entropy of the input probabilities (gray horizontal dashed line). These results show that as the amount of homogenization relative to competition increased, the rate of change of synaptic modification decreased. Together, the results of these analyses demonstrate that the entropy of the final synaptic weight distribution is strongly influenced by the balance of competitive and homogenizing forces expressed by HCP.

### Encoding backward probabilities from experienced forward probabilities

The results described above were for simulations in which the network experienced the forward conditional probabilities of BF song sequences, and used HCP with pre-synaptic competition. This engrained the forward conditional probabilities into the synaptic weight matrix M connecting the nodes in the network. As described in the Introduction, one of the motivating examples for the present study was our observation of a nearly linear encoding of the backward conditional probabilities in the auditory responses of HVC neurons (Bouchard and Brainard, 2013). Thus, we additionally examined learning backward probabilities using the same network architecture, learning rule and sequence statistics described above, with the only difference being that post-synaptic competition was induced by divisively normalizing the sum of post-synaptic weights.

**Figure 7:**
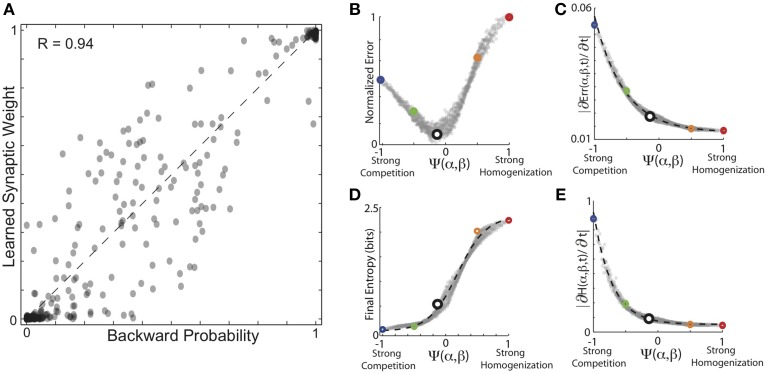
**Encoding of backward probabilities from experienced forward probabilities**. **(A)** Conditional backward probabilities vs. learned synaptic weights resulting from HCP with optimal values of α and β. Here, each point corresponds to one transition/weight pairing (*n* = 1018 transitions). Dashed line is unity. **(B)** Average normalized error as a function of the homogenization-competition ratio Ψ(α, β) (see text). Coloring correspondence same as in Figures 5, 6. **(C)** Magnitude of initial error reduction (∂Err(Ψ,t)/∂t) generally decreased as a function of increasing the magnitude of the homogenizing force. **(D)** Average final entropy of the synaptic weight matrices as a function of Ψ. Black dashed line is best fitting hyperbolic tangent, which explains 99% of the variability (*R*^2^ = 0.99) in final entropy as a function of Ψ. **(E)** Magnitude of initial entropy reduction (∂H(Ψ,t)/∂t) decreased as a function of increasing Ψ.

As expected, overall, the results for learning backward probability and forward probability exhibit similar dependencies on the balance of competitive and homogenizing forces expressed by HCP. The results of these simulations are summarized in Figure 7. In Figure 7A, we plot the conditional backward probabilities for the *n* = 1018 transitions from 16 birds used here vs. the learned synaptic weights resulting from HCP with optimal values of α and β. Here we see that the backwards probabilities were learned with a similar degree of fidelity to the forward probabilities, but with a slightly reduced accuracy (*R* = 0.94 for backward probabilities vs. *R* = 0.97 for forward probabilities). The plots in Figures 7B,C display the average final error and rate of initial error reduction as a function of function Ψ(α, β). The plots in Figures 7D,E display the average entropy and rate of initial entropy reduction as a function of function Ψ(α, β). These curves are very similar to those presented in Figures 5, 6 in many regards. The most salient difference is the reduced normalized error for learning backward probabilities at very negative values of Ψ relative to learning the forward probabilities. This small difference likely results from the differences in distributions between forward and backward probabilities (Bouchard and Brainard, 2013). We note that if firing rates are a linear function of synaptic weights, the synaptic weights generate neural responses that increase linearly as function of backward probability, qualitatively matching the experimentally observed auditory responses in Bouchard and Brainard (2013).

### The optimal magnitude of homogenizing force depends on the signal-to-noise ratio in the network

**Figure 8:**
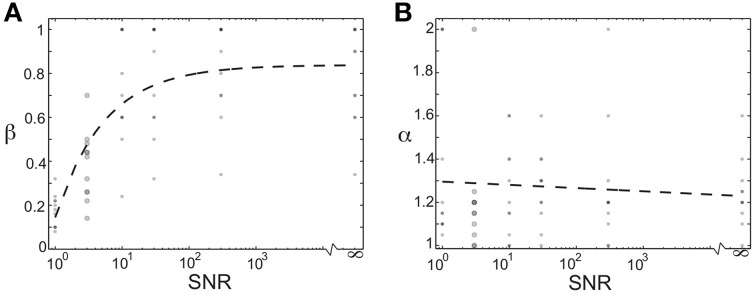
**The optimal magnitude of homogenizing force depends on the signal-to-noise of the network. (A)** Optimal value of β as a function of the signal-to-noise ratio in the network. Each dot corresponds to the value derived from one of 11 birds used here (subset of the 16 used in this study). Dashed black line is best fitting exponential function. Large dots correspond to SNR used for main results. **(B)** Optimal value of α as a function of the signal-to-noise ratio in the network. Each dot corresponds to the value derived from one of 11 birds used here (subset of the 16 used in this study). Dashed black line is best-fitting line. Large dots correspond to SNR used for main results.

We additionally investigated how the optimal values of the homogenizing force (β) and competitive force (α) depended on the relative amount of noise in the teacher signal. We modulated the signal-to-noise ratio of the network by changing the magnitude of the Poisson noise in each node in the network from (on average) the same size as the activation signal (SNR = 1) to no noise (SNR = ∞). The plots in Figures 8A,B show the optimal values of homogenizing force (β) and competitive force (α) as a function of increasing signal-to-noise ratio. Each dot corresponds to the average values for one of 11 birds (a subset of the 16 total used in this study; overlap of points indicated by opacity). The large dots demarcate regime used in Figures 4–7; for the other SNRs, a coarser spacing was used in the grid search. Here, we see that increasing the signal-to-noise ratio resulted in higher optimal values of (β), with SNRs over 10 requiring very large homogenization terms (Figure 8A). Furthermore, even for an SNR of 1, optimal values of β were around 0.2, suggesting that even in this case, homogenizing forces played an important role in stable learning (though strong conclusions cannot be drawn because of potential edge effects in the range of this parameter). In contrast, the optimal values of α did not change in a consistent manner for varying SNR. This suggests that the magnitude of homogenizing force expressed by Hebbian learning rules required to learn a given weight distribution depends on the amount of noise in the network.

### The optimal balance of competitive and homogenizing forces depend on the entropy of the target distribution

**Figure 9:**
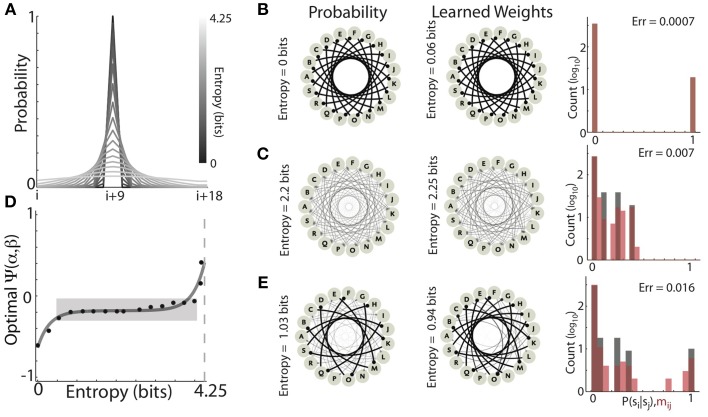
**The optimal balance of competitive and homogenizing forces depend on the entropy of the target distribution**. **(A)** Profile of the Gaussian forward transition probabilities (19 states, centered at the i+9 state) for varying standard deviations. As the standard deviation (entropy) of the distributions increases, the gray scale lightens. **(B,C)** Examples of learning Gaussian forward probabilities with different entropies. Both low entropy (0 bits) **(B)** and medium entropy (2.2 bits) **(C)** could be well learned with different values of the homogenizing force (β) and the competitive force (α) expressed by HCP. The graphs on the left are depict the forward probability diagrams (nodes are states, edges are transitions), while the graphs in the center depict the synaptic connectivity of the learned network (nodes are network elements, edges are synaptic connections). The histograms on the right show the distributions of forward probabilities (black bars), and learned synaptic weights (red bars). **(D)** The optimal value of Ψ exhibited a non-decreasing dependence on the entropy of the target forward transition matrix. The hyperbolic sine (sinh, dashed black line) dependence of optimal Ψ on target entropy explained 93% of the observed variability (*R*^2^ = 0.93). Dashed light gray line is maximum entropy for a 19 state matrix (~4.26 bits). Approximately 80% of the distributions could be well learned with homogenization and competition values that occupied only ~10% of the range of Ψ (gray shaded region). **(E)** Learning transition matrices composed of mixed distributions (in this case alternating the 0 bit and 2.2 bit distributions) resulted in increased learning error relative to learning the individual distributions.

We have shown that optimal learning of conditional probability distributions by Hebbian learning requires a balance of competitive and homogenizing forces. These forces, in combination with the statistics of the target matrix, dictate the entropy of the steady-state synaptic weight distributions. Indeed, the results presented in Figures 6B, 7D suggest that to achieve optimal learning, the relative strength of homogenization should increase as the entropy of the target distribution increases. In the most extreme case, it could be that optimal learning requires the relative magnitudes of these forces to be finely tuned to the statistics of the target distribution. Such a situation would suggest that neural systems must maintain a very delicate balance of these forces, which would potentially reduce their robustness. To systematically investigate how the optimal balance of α and β depended on the entropy of target transition probability distributions, we again used Gaussian transition matrices with 19 states. The recurrent network architecture and inputs are the same as before (Figure 3A). We varied the entropy of the forward probability distributions by steadily increasing the standard deviation of the Gaussian distribution. The gray curves in Figure 9A display the family of distributions used, which range from completely deterministic (P_F_ = 0 or 1, Entropy = 0 bits, black) to an approximately uniform distribution (P_F_ ~ 1/19, Entropy = 4.25 bits, lightest gray).

We found that, as the entropy of the target distribution increased, so did the magnitude of homogenization relative to competition required for optimal learning. Two examples of learning Gaussian forward probabilities with different entropies are displayed in Figures 9B,C. In Figure 9B, the forward probabilities of the experienced transitions (left) had 0 bits of entropy and the final synaptic weights of the network (right) was a near perfect copy (Entropy = 0.06 bits, Error = 0.0007). Optimal learning in this condition expressed a strongly competitive tone in HCP (α = 1.9, β = 0.18, Ψ = −0.61). In Figure 9C, the forward probabilities of the experienced transitions were more variable (2.2 bits of entropy) and the learned recurrent weight matrix was again a very good match (Entropy = 2.25, Error = 0.007). Optimal learning in this condition required a stronger homogenizing tone in HCP (α = 1.05, β = 0.34, Ψ = −0.17).

Across the range of distributions tested, optimally tuned HCP was able to mold the synaptic weights to be a close approximation of the experienced forward probabilities, with an average error of 0.0066 ± 0.0026 (mean ± s.d.). The optimal balance of competition and homogenization exhibited a systematic dependence on the entropy of the forward probability distribution (Figure 9D, the dashed black line is the best fit hyperbolic sine (sinh), *R*^2^ = 0.93). Associating negative values of Ψ with strong competition and positive values of Ψ with strong homogenization, these results show that distributions with low entropy required relatively stronger competitive tone while distributions with high entropy required relatively stronger homogenizing tone (Figure 9D). Thus, the optimal strength of homogenization increased as the entropy of the experienced transition probabilities increased. However, it was not the case that the relative amounts of homogenization and competition had to be finely tuned for each distribution. Strikingly, ~80% of the distributions were optimally learned with homogenization-competition values that occupy ~10% of the range of Ψ (gray shaded region in Figure 9D). Furthermore, all but the two most entropic distributions resulted in optimal Ψ values less than 0, showing that, in general agreement with the results from Bf sequences (Figures 5, 7), accurate Hebbian encoding of sequence probability requires β < 0.5. Thus, HCP operating within a limited regime of homogenizing and competitive forces is capable of learning the vast majority of probability distributions tested.

The systematic dependence of Ψ required to optimally learn transition probability distributions of increasing entropy suggested that learning transition matrices with a mixture of low and high entropy transitions should be problematic. The example shown in Figure 9E provides a demonstration of such a case. Here, the entropy of transition probabilities alternated between 0 (Figure 9B) and 2.2 bits (Figure 9C). Optimal learning in this case resulted in a larger learning error than either of the two pure distributions (Error = 0.016) and the final average entropy (Entropy = 0.94 bits) was considerably less than the average entropy of the input sequences (Entropy = 1.03 bits). These results provide insight into the origin of deviations from a perfect encoding observed for learning Bf songs in Figures 5A, 7A. In our simulations, the parameters α and β were jointly optimized to minimize the average error across all transitions for all syllables simultaneously. Therefore, because the song sequences of an individual Bengalese finch can exhibit a large range of forward entropies across different syllables (with a bias toward low-entropy distributions), these results suggest that a single α and β that are optimal across all transitions may not exist for these sequences. In agreement with this, across the transition matrices for the 16 birds examined here, we observed that average learning error was positively correlated with the variability in the entropy of individual syllable transitions (*R* = 0.52, *P* < 10^−7^) (Equation 8).

### Balanced spike-timing dependent plasticity (STDP) learns conditional probability of spiking activity

**Figure 10:**
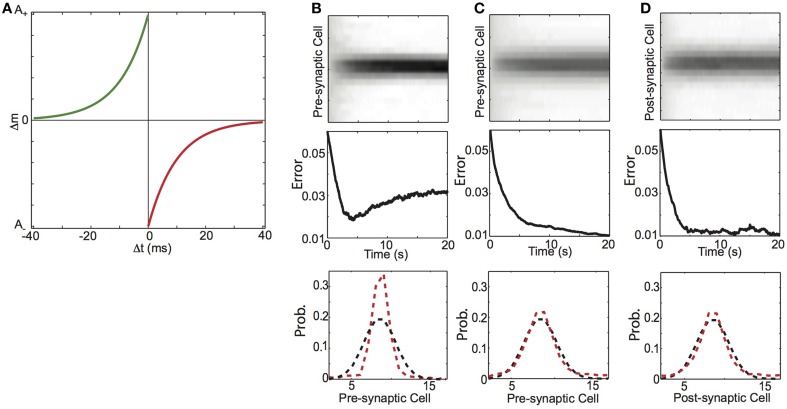
**Balanced spike-timing dependent plasticity (STDP) learns conditional probability of spiking activity. (A)** Spike-timing dependent plasticity rule. The amount of synaptic change depends on the temporal difference (Δt) between pre and post-synaptic spike times. Synaptic potentiation (green curve) decays exponentially from a maximum magnitude of A_+_as the difference between pre-synaptic spike preceding post-synaptic spikes (−Δt) increases. Conversely synaptic depression (red curve) decays exponentially from a maximum magnitude of **(A)** as the difference between post-synaptic spike preceding pre-synaptic spikes (Δt) increases. **(B–D)** Learning conditional probabilities with STDP. **(B)** Additive STDP, **(C)** Balanced STDP, **(D)** Balanced STDP with pre-synaptic normalization. (top) Synaptic weights (gray scale) as a function time. (middle) Difference between synaptic weight distribution and the underlying conditional probability of a presynaptic spike preceding a post-synaptic spike. (bottom) Probability distribution of final weight vector (red) and underlying probability distribution (black).

The results above demonstrate that Hebbian plasticity develops synaptic weights equal to conditional probabilities. We next investigated whether such results hold under more biologically realistic conditions. To this end, we again considered a feed-forward network architecture of a single post-synaptic neuron (y) connected to pre-synaptic neurons (x_j_) through weights m_j_. The network is activated by an input sequence such that during some plasticity window (Δt), x_j_ and y are sequentially activated with probability P(x_j_|y) (Figure 2A). This time, units are modeled as conductance based, leaky integrate-and-fire units (using generic parameters) with Poisson background spikes (Methods). In addition to the background Poisson spiking, probabilistic sequential spiking is imposed on the pre-synaptic and post-synaptic neurons through large excitatory conductances (the “teaching” input to the network). Specifically, a spike is imposed on a pre-synaptic neuron according to a Gaussian distribution over the identity of pre-synaptic cells and 5 ms later (Δt = −5 ms), a spike is generated in the post-synaptic cell. We used standard temporally asymmetric Hebbian plasticity based on spike timing (spike-timing dependent plasticity, STDP), to modify the synaptic weights (Figure 10A). These simulations test the specific hypothesis that STDP molds synaptic weight distributions to reflect the conditional probability of spike sequences experienced by a feed-forward network.

According to the STDP rule, the change in synaptic strength between a pre-synaptic and post-synaptic neuron (Δm_j_) decays exponentially (here, with equal decay rates τ_±_) as the temporal difference in their spike times (Δt) increases (Figure 10A) (Kistler and van Hemmen, 2000; Song et al., 2000; Kempter et al., 2001; Rubin et al., 2001; Kepecs et al., 2002). Synapses are potentiated if the pre-synaptic neuron spikes with some short latency before the post-synaptic neuron, and are depressed if post-synaptic spikes precede pre-synaptic spikes (Figure 10A) (Equation 9). As with HCP, A_±_ describe the magnitude of synaptic change (learning rates) and f_±_(m_j_) are functions that describe how the magnitude of synaptic change depends on the current synaptic weight (Song et al., 2000; Kempter et al., 2001; Rubin et al., 2001; Gütig et al., 2003). The temporal asymmetry in STDP has been interpreted as organizing potentiation and depression to reflect the degree to which pre-synaptic spikes caused post-synaptic spikes (Sjöström et al., 2001; Dan and Poo, 2004).

We found that STDP with a modest depression/potentiation ratio (α = 1.1) and no weight dependence of synaptic change (β = 0, the “additive” STDP rule), did not shape synaptic weights to reflect the forward conditional probability of spiking. The top plot of Figure 10B shows the synaptic weights over 20 s of simulation (corresponding to 1000 imposed spike pairs), and we see that the weight distribution steadily becomes more concentrated on the neurons that have the highest transition probability at the expense of the less probable neurons. This results in an initial reduction in the error (mean absolute difference) between weight and transition probability distributions, which then increased after ~5 s (250 imposed spike pairs) (Figure 10B, middle). The final weight distribution (Figure 10B, bottom, red) is more sharply peaked than the underlying conditional probability distribution (Figure 10B, bottom, black). In contrast, we found that STDP with the same depression/potentiation ratio (α = 1.1) and a modest amount of weight dependence of synaptic change (β = 0.2), could shape synaptic weights to reflect the backward spiking conditional probability. In Figure 10C, we show that such “balanced STDP” was able to maintain the values of small synaptic weights (top), with learning dynamics that decayed monotonically (middle), resulting in a synaptic weight distribution that is very well matched to the underlying conditional spike probability distribution (bottom).

To next examine if STDP can encode forward probabilities we again considered a feed-forward network architecture of a single pre-synaptic neuron (y) connected to post-synaptic neurons (x_j_) through weights m_j_. The neurons are activated as described above. In line with our HCP learning rule and previous modeling work (Fiete et al., 2010), we augmented the standard STDP rule (Figure 10A), by divisively normalizing pre-synaptic weights. As shown in Figure 10D, this learning rule rapidly shapes the synapses from the pre-synaptic neuron on to the post-synaptic targets to reflect the conditional probability with which they were active. Together, these results demonstrate that, with an appropriate balance of homogenizing and competitive forces, STDP in a feed-forward network can engrain either the forward or backward probability.

## Discussion

We have studied the properties that enable a variety of Hebbian learning rules (correlation, HCP, STDP) to engrain conditional Markov transition probabilities in the synaptic weights of sequentially active networks. We found that Hebbian plasticity rules with pre-synaptic competition resulted in synaptic weights that reflected the forward probabilities of the input sequences while post-synaptic competition resulted in synaptic weights that reflected the backward probabilities of the same input sequence. Furthermore, to accurately and stably reflect the statistics of input sequences, Hebbian plasticity (STDP and HCP) required a balance of the competitive and homogenizing forces expressed by plasticity rules. Together with the statistics of the inputs, the balance of these forces strongly influenced both the entropy of the steady-state synaptic weight distribution as well as the rate at which structure emerged. We demonstrate that the optimal balance of these forces depends on the entropy of the probability distribution to be learned; however, a large proportion of distributions could be learned within a small range of parameters. Together, these results reveal properties of Hebbian plasticity that allow neural networks to encode conditional probabilities of sequences.

### Learning forward and backward probabilities with pre and post-synaptic competition

The geometry of many neurons exhibits a pronounced asymmetry between their dendrites and axons, and this physical asymmetry suggests an asymmetry in information processing (Abeles, 1991; Dayan and Abbott, 2001; Koch, 2004). The distribution of post-synaptic weights describes a neuron's receptive field (i.e., how information flowing into the neuron affects its activity), while the pre-synaptic weights describe a neuron's projective field (i.e., how information flows out of the neuron) (Abeles, 1991; Dayan and Abbott, 2001; Koch, 2004). Based on these functional/anatomical considerations and previous theoretical studies, we hypothesized that Hebbian plasticity with a post-synaptic locus of competition would develop weight distributions that reflect the backward probabilities of the input sequence, and conversely, that Hebbian plasticity with a pre-synaptic locus of competition would develop weight distributions that reflect the forward probabilities of the same input sequence (Grossberg, 1969; Amari, 1972; Miller and MacKay, 1994; Fiete et al., 2010).

Our results demonstrate that the synaptic weights of neural networks will come to reflect different probabilistic structures of the same input sequence depending on the site of synaptic competition. We show that when synaptic competition is instantiated pre-synaptically the weights converge on the forward conditional transition probabilities. Conversely, when competition is instantiated post-synaptically the synaptic weights converge to the backward conditional transition probabilities of the same sequence. These two probabilistic structures have different semantics attached to them. The forward probability describes the future of the system; it is the probability of transitioning *from* the current state *to* any state. In contrast, the backward probability describes the past of the system; it is the probability of transitioning *to* the current state *from* any state. From a functional standpoint, synaptic encoding of forward probabilities are not only potentially useful for producing sequences with a given probabilistic structure (Jin, 2009), but also to make predictions about upcoming (sensory) events (Bastos et al., 2012). The backward probabilities, on the other hand, are useful for recognition processes, among other things (Gentner and Margoliash, 2003; Bouchard and Brainard, 2013). The specifics of how synaptic weights proportional to probabilities get used for these different functions depend on the precise mechanism by which the weights are transformed into neuronal activities. No matter how weights are transformed to outputs for specific functions, our results demonstrate an important and remarkably simple correspondence between the biophysical organization of neurons, the site of synaptic competition, and the temporal flow of information encoded in synaptic weights by Hebbian plasticity (Grossberg, 1969; Miller and MacKay, 1994; Dayan and Abbott, 2001; Koch, 2004).

Previous studies in humans and birds have demonstrated neural responses that increase (linearly) with increasing conditional probabilities of experienced vocal sequences (Bouchard and Brainard, 2013; Leonard et al., 2015). For neurons operating in a linear regime, synaptic weights that are linearly proportional to experienced probabilities would generate auditory responses to sequence playback that linearly increases with increasing probability. Therefore, intuitively, the networks trained here could qualitatively re-produce previously published results in humans and birds described above. We note that the Hebbian encoding of sequence probability observed here, in which weights are directly proportional to conditional probabilities, is at odds with the most basic formulations of efficient/predictive coding theories (Barlow, 1961; Bastos et al., 2012). These powerful theories generally predict that neural responses should encode sensory “surprise,” and thus be inversely proportional to experienced probability (e.g., in “odd-ball” experiments). We note that for both humans and birds, the statistics of vocal sequences (speech and birdsong, respectively) are highly behaviorally relevant (Bouchard and Brainard, 2013; Leonard et al., 2015), and are learned over long time scales. Our study supports the hypothesis that the neural responses observed in these studies can emerge through local Hebbian plasticity operating on the experienced statistics of ethologically important sensory sequences.

Recent modeling studies that have aimed to generate connectivity matrices capable of producing unary chains of activity (i.e., linear sequences) from initially random connectivity have incorporated some form of pre-synaptic plasticity (Jun and Jin, 2007; Fiete et al., 2010). While these previous studies suggested an important role for pre-synaptic plasticity in forming weight matrices capable of generating linear sequences, the precise statistical structure being captured by this plasticity was not examined. Of particular relevance to our work are the results of Fiete et al. (2010), which showed that connectivity matrices capable of generating unary chains of spiking activity in recurrent neural networks (i.e., linear sequences) can emerge spontaneously from an initially randomly connected matrix. For this result, the crucial components of the learning algorithm were a temporally asymmetric Hebbian plasticity rule combined with constraints on both the sum of pre-synaptic and the sum of post-synaptic weights (Fiete et al., 2010). As shown here, constraining the sum of the pre-synaptic weights generates pre-synaptic competition, while constraining the sum of the post-synaptic weights generates post-synaptic competition. In the context of Fiete et al, constraining both presynaptic and post-synaptic weighs during STDP creates a highly competitive, essentially “winner-take-all” regime, giving rise to the completely unary chain of synaptic connectivity. Our results demonstrate that a specific probabilistic representation, conditional forward probability, emerges through pre-synaptic competition. This emphasizes the importance of pre-synaptic competition in formation of synaptic weight matrices that underlie behavioral sequence generation and prediction in neural networks.

### Neurobiological mechanisms of pre-and post-synaptic competition

The importance of heterosynaptic competition hypothesized by modeling studies raises the question of the biophysical plausibility of this type of synaptic modification. There is both electrophysiological and structural evidence supporting the hypothesis that neurons conserve the total synaptic weight they receive, which could induce heterosynaptic competition post-synaptically (Abraham et al., 1985; Royer and Paré, 2003; Bourne and Harris, 2011). Of particular relevance is the observation that when one group of synaptic inputs to a neuron is potentiated through activity dependent homosynaptic LTP, the other synapses can undergo heterosynaptic LTD (Royer and Paré, 2003). In this way, the sum of synaptic inputs to the neuron is held constant (Royer and Paré, 2003). This is direct electrophysiological evidence for heterosynaptic competition induced by the conservation of post-synaptic weights, as used here for HCP.

On the pre-synaptic side, the existing experimental literature supports the possibility of heterosynaptic competition/plasticity expressed pre-synaptically. It has been shown that axonal terminals originating from the same pre-synaptic neuron can vary their synaptic strength depending on the post-synaptic target, demonstrating the ability to regulate pre-synaptic strength (Koester and Johnston, 2005). Activity dependent homosynaptic plasticity of existing synapses has been observed to be pre-synaptically expressed at several synapses, including bi-directional plasticity at the CA3 to mossy-fiber synapse in the mammalian hippocampus (Nicoll and Schmitz, 2005) and in aplysia (Bailey et al., 2004), and LTD at L4 to L2/3 synapses in the barrel cortex (Bender et al., 2006a,b). Additionally, activity dependent, Hebbian competition of axonal territory and synaptic strength has been observed for retino-tectal projection (Ben Fredj et al., 2010; Munz et al., 2014). Finally, strong evidence exists for a pre-synaptic site of expression underlying activity dependent homeostasis at the Drosophila neuro-muscular junction (Frank et al., 2006) and in dissociated hippocampal (Branco et al., 2008) and cortical cultures (Turrigiano et al., 1998). Together, these results support the neurobiological plausibility of heterosynaptic competition through constraints on the total out-going weight of a pre-synaptic neuron. A key testable physiological prediction is that Hebbian protocols for inducing synaptic plasticity (e.g., STDP) between a single pre-synaptic neuron contacting two (or more) independent post-synaptic neurons should conserve the total magnitude of synaptic potentials across post-synaptic neurons.

### Balancing synaptic homogenization and competition to match statistics

Independent of the site of synaptic competition, we found that optimal learning of conditional probabilities by biologically inspired Hebbian plasticity rules required a balance of the weight dependence of synaptic change and the relative magnitudes of depressing and potentiating events. Competitive forces push synapses toward the binary synaptic weight distribution (i.e., low entropy), while homogenization pulls synapses toward the uniform synaptic weight distribution (i.e., maximal entropy). Therefore, the difference in the initial dynamics of learning as a function of the amount of homogenization/competition can be understood in terms of the stability of the initial weight matrices (M_t=0_ ~ 1/n) under the forces induced by the various amounts of homogenization and competition. The weight dependence of synaptic change and the depression-potentiation ratio did not operate independently, as we found that the magnitude of the effects mediated by either α or β was modulated by the other (Figures 5B, 6A). As competition and homogenization are, at a very basic level, antagonistic forces, an interaction between the two is to be expected. We note that the observed dependencies of accuracy and speed of learning on these forces is not simply a reformulation of a “speed-accuracy trade-off,” as we found that accuracy depended non-monotonically on Ψ while learning rate decreased in an approximately monotonic fashion. Interestingly, it has recently been shown that the detailed shape of the STDP window function near the transition from depression to potentiation is critical in determining the consequences of STDP (Babadi and Abbott, 2010). This may serve as a biophysical mechanism for shifting the balance of homogenizing and competitive forces, and is an interesting direction of future research.

In an elegant paper using a similar training paradigm to ours, Legenstein, Naeger, and Maas have proven that through post-synaptic STDP, a neuron can implement a wide variety of transformations of its inputs (Legenstein et al., 2005). A recent study on reward based synaptic plasticity has shown that post-synaptic plasticity can engrain posterior probabilities of decisions based on cues given rewards (Soltani and Wang, 2010). However, the plasticity rule was not based on activity levels, and long-term instabilities in weights can be observed in the simulations (Soltani and Wang, 2010). Our work suggests that equipping this rule with parameters to balance homogenizing and competitive forces would stabilize its long-term behavior. Specifically, our results show that Hebbian plasticity expressing a balance of homogenizing and competitive forces resulting from a limited range of parameters (here, ~10% of the parameter space) is capable of stably learning the vast majority of distributions to which it is exposed (here, ~80%). This suggests that a nervous system with Hebbian plasticity operating within this regime would be well poised to learn all but the most extreme statistical distributions it encounters. However, the converse of this is also true, namely that the balance of these forces dictates a prior expectation of the randomness of the sequence to be learned. Therefore, in the context of sequence learning, the balance of these forces may be tuned through evolution to bias the sequences of one species to be different from other species. For example, in songbirds the zebra finch sings a nearly deterministic sequence of syllables, while the closely related Bengalese finch (which served as our ethological data set) exhibits a song with complex probabilistic sequence structure. Hence, a specific prediction is that over generations, the sequences produced by a given birdsong species will converge to a common entropy value, set by the biophysical properties of synaptic plasticity for that species. At the same time, some of the variability observed in the accuracy of biological sequence learning may result from individual differences in the balance of the homogenizing and competitive forces, possibly creating a mismatch between intrinsic learning parameters and the statistics of the sequence to be learned. Thus, like all priors, the prior dictated by the balance of homogenization and competition is a “double-edged sword,” enhancing learning when experiences matches its structure, but hindering learning when experiences deviate from it.

Together with previous modeling and analytic work, our study illustrates the importance of understanding the neurobiological processes that give rise to the competitive and homogenizing forces expressed by synaptic plasticity (Miller and MacKay, 1994; Miller, 1996; Song et al., 2000; Rubin et al., 2001; Sjöström et al., 2001; Kepecs et al., 2002; Gütig et al., 2003; Legenstein et al., 2005; Babadi and Abbott, 2010). What balance of homogenization and competition does synaptic plasticity in the nervous system express? Is this balance specific to a given brain region? Does the site of synaptic plasticity depend on the computation required of the network? The answer to these questions awaits further experimental work. For example, the development of ocular dominance columns requires connectivity matrices that are essentially binary, suggesting that the Hebbian mechanisms underlying this development exhibit a relatively strong competitive tone (Miller et al., 1989; Song and Abbott, 2001). In contrast, the distribution of EPSC's measured experimentally from a variety of brain areas (Sayer et al., 1990; Mason et al., 1991; Sjöström et al., 2001; Feldmeyer et al., 2002; Barbour et al., 2007) seems to suggest a more multiplicative type rule (van Rossum et al., 2000; Babadi and Abbott, 2010). Together, these results suggest that the amounts of homogenization and competition expressed by Hebbian mechanisms may vary from area-to-area, or maybe developmentally regulated, depending on the “desired” structure of the weight matrix. For example, the development of sparse neural representations, which require the majority of feature encoding weights to be 0, may be aided by strongly competitive Hebbian plasticity, while more dense representations could emerge from strongly homogenizing Hebbian plasticity (Olshausen and Field, 1996; Perrinet, 2010; Ganguli and Sompolinsky, 2012). Put another way, the balance of homogenizing and competitive forces dictates a prior expectation of the distribution of the synaptic weights to be learned, and therefore the computational goal of the circuit should constrain these forces (Legenstein et al., 2005; Barbour et al., 2007).

### Conflict of interest statement

The authors declare that the research was conducted in the absence of any commercial or financial relationships that could be construed as a potential conflict of interest.
